# Assessment of Knowledge, Attitude, and Practice of Hand Hygiene Among Medical Students in a Tertiary Care Hospital

**DOI:** 10.7759/cureus.66820

**Published:** 2024-08-13

**Authors:** Nandhini Ravella Venkatasubramanyam, Abiramasundari Vadivel Kalyanasundaram, Neelusree Prabhakaran

**Affiliations:** 1 Department of Microbiology, Saveetha Medical College and Hospital, Saveetha Institute of Medical and Technical Sciences, Saveetha University, Chennai, IND

**Keywords:** hand disinfectants, kap survey, hand hygeine, knowledge-attitude-practice theory, knowledge assessment

## Abstract

Introduction: Healthcare-associated infections (HCAIs) and emerging multi-drug resistance in nosocomial pathogens are perceived as a serious public health threat. Hands of healthcare workers (HCWs) become routinely colonized during patient care, serving as vehicles for transmission and leading to HCAIs. Hand hygiene (HH) is a globally accepted tool to avoid the broadcast of dangerous microorganisms and prevent HCAIs.

Materials and methods: A cross-sectional study was carried out to evaluate the knowledge, attitude, and practice toward HH among the medical students at Saveetha Medical College, Chennai, India. A 25-item validated questionnaire survey was formulated and circulated to 100 medical students of all four academic years.

Results: There were 100 responses to the survey, and 44 (44%) participants performed HH appropriately for 20 seconds with alcohol-based hand rub. In our study, 25％ of the participants revealed that HH practices were not followed during emergencies. Many participants (40%) stated that the lack of sink, soaps, alcohol-based sanitizers, paper towels, and water is the reason for not performing HH.

Conclusions: The gross knowledge of HH of the participants is moderate, but there were gaps between the knowledge and practice. Hence, it is essential to conduct structured training sessions and surveillance programs for medical students to address these gaps in knowledge and the correct HH procedures.

## Introduction

Healthcare-associated infections (HCAIs), also known as nosocomial infections, are infections acquired by patients during their hospital stay or even after discharge, which were not present upon admission, as well as occupational infections among healthcare workers (HCWs) within the facility. The occurrence rate of HCAIs in developed nations ranges from 5.1% to 11.6%, whereas in developing countries, this rate can soar up to 19%, positioning these infections among the top 10 causes of death related to hospital care. Numerous factors contribute to the incidence of HCAIs, including but not limited to inadequate hand hygiene (HH), extended stays in hospitals, complex medical procedures, long-term disabilities, improper use of medical devices, cross-infection, antimicrobial-resistant pathogens, and compromised immune systems of patients [1,2]. Globally, HCAIs are a significant cause of illness and death, with human hands being the primary vehicle for the transmission of germs in all healthcare settings [3].

HH is identified as the most crucial action to prevent the cross-transmission of harmful microorganisms and to decrease the prevalence of HCAIs [4]. The practice of HH, involving the use of alcohol-based hand rub for 20 to 30 seconds or washing hands with soap and clean running water for 40 to 60 seconds, is highly effective in combating pathogens responsible for HAIs, including those that are multidrug-resistant (MDR) [5]. This hygiene protocol includes cleansing hands prior to patient contact, before conducting sterile and non-sterile tasks, after potential exposure to pathogens, after interacting with a patient, and after contact with objects in the patient’s environment. Following current CDC guidelines, the use of alcohol-based hand sanitizers is advised when hands are not visibly dirty, and washing with soap and water is recommended when hands are visibly contaminated with bodily fluid [5,6]. Maintaining optimal HH among healthcare practitioners is pivotal in preventing HCAIs [7]. Despite the simplicity of HH practices, adherence among healthcare providers is alarmingly low, around 40%, presenting a global health concern [8]. Factors influencing poor compliance with HH among HCWs include workload, time constraints, lack of knowledge, negative attitudes, and misconceptions about HH and infection control practices. In addition, healthcare facilities might face challenges like inadequate HH resources, lack of supervision, insufficient training, and the absence of influential role models in promoting compliance. In response, ongoing efforts are dedicated to finding effective and lasting solutions.

The World Health Organization (WHO) has classified HCAIs as one of the leading causes of hospital mortality globally and has introduced a pivotal strategy known as “My five moments for hand hygiene'' [9,10]. Implementing preventive measures to break the chain of infection transmission is critical, with HH standing out as the most effective and economical option. Nonetheless, a significant gap in HH knowledge persists among healthcare students and professionals. Anyone involved in direct or indirect patient care must prioritize HH and perform it correctly and timely. It is estimated that around 4% of acute care hospital admissions result in one or more nosocomial infections, affecting 5-15% of hospitalized patients in developed countries [11]. Enhancing the quality of healthcare services through the strict implementation of HH practices is essential, requiring structured training and surveillance initiatives. This study aims to provide standardized data on the knowledge, attitudes, and self-reported practices concerning HH among undergraduate medical students to identify educational gaps and evaluate factors influencing their HH behaviors.

## Materials and methods

This cross-sectional study was conducted at Saveetha Medical College, a tertiary care hospital with over 1600 beds in Chennai, Tamil Nadu, India, from June 2023 to August 2023. Before the commencement of the study, approval was obtained from the Institutional Ethical Review Committee of Saveetha Medical College (IEC-Reference Number: 163/06/2023/IEC/SMCH), ensuring that all research protocols adhered to ethical standards. The study population consisted of 100 medical graduates who voluntarily agreed to participate in the research. These individuals were fully informed about the nature and objectives of the study, after which verbal consent was obtained from each participant, aligning with ethical research practices. The research tool utilized in this study was a self-administered questionnaire, which was composed of a total of 25 items. These items included 12 questions assessing knowledge and 13 questions evaluating attitudes and practices, which were derived from a questionnaire previously developed by the World Health Organization (WHO) for HCWs. The questions were designed to elicit responses in various formats, including "yes" or "no" answers and multiple-choice options for the assessment of knowledge. Attitudes were gauged through 10 statements to which respondents could express agreement or disagreement. Practices were similarly evaluated using seven questions. Each question was designed to objectively measure the respondent's knowledge, attitudes, and practices related to the study's focus area. A scoring system was employed to quantify the responses, where each correct answer or positive response was awarded one point. Incorrect or negative responses were assigned zero points. The cumulative scores were then categorized into three levels of proficiency: a score above 75% was classified as good, scores ranging from 50% to 74% were considered moderate, and scores below 50% were deemed poor.

Data analysis

Data were collected and entered into Microsoft Excel (Microsoft Corporation, USA) and then analyzed using IBM SPSS Statistics for Windows, Version 25.0 (released 2017, IBM Corp., Armonk, NY). Descriptive statistics were used to calculate the frequency and percentage. This analysis facilitated the identification of significant patterns, trends, and correlations within the data, contributing to the overall findings and conclusions of the study.

## Results

**Figure 1:**
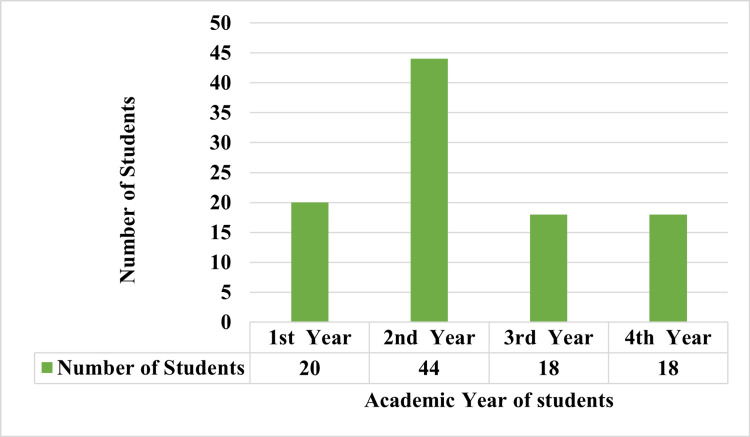
Distribution of respondents by academic year

The study conducted at Saveetha Medical College, Chennai, involved 100 medical students from various years of their medical education. The distribution of participants across different academic years is illustrated in Figure 1. The second-year medical students comprised nearly half of the participants, accounting for 44% of the total respondents. First-year students made up 20%, while third and fourth-year students each represented 18% of the respondents.

**Figure 2:**
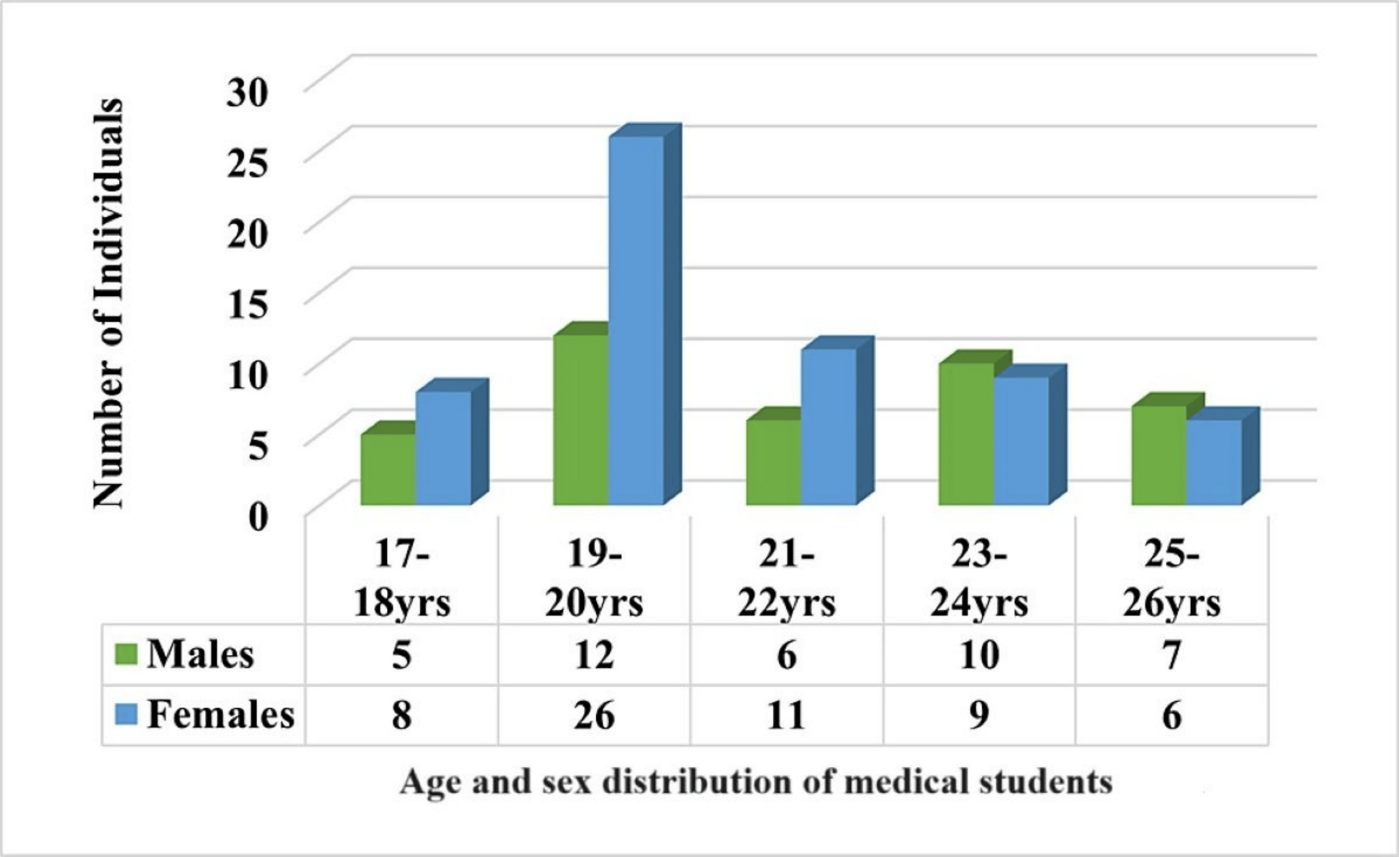
Age and sex distribution of medical students.

The age and sex distribution of medical students as presented in Figure 2. The data show a total of 100 medical students categorized into five age groups ranging from 17 to 26 years. The youngest age group, 17-18 years, consists of 13 medical students, with females (eight) outnumbering males (five). This trend of females outnumbering males continues in the 19-20 years age group, which has the highest total number of students (38), with females (26) significantly outnumbering males (12). The 21-22 years age group shows a more balanced distribution, with 11 females and six males, totaling 17 students. The 23-24 years age group has a near-equal gender distribution, with 10 males and nine females, summing up to 19 students. Finally, the 25-26 years age group sees a reversal in the earlier trend, with males (seven) slightly outnumbering females (six), totaling 13 students.

**Figure 3:**
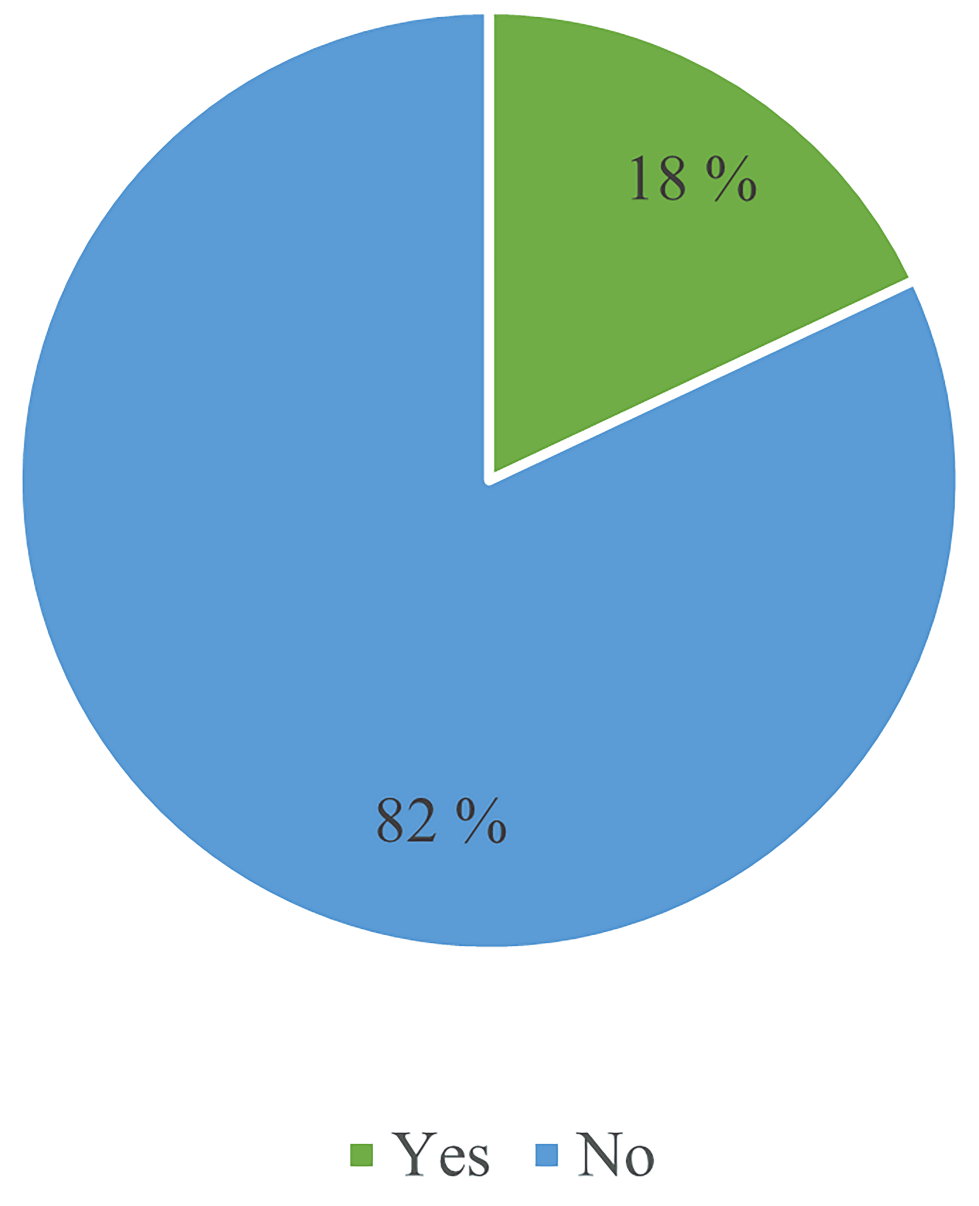
Training received in hand hygiene.

The data on HH training among a group of 100 medical students reveals that only 18% (n = 18) reported having received training in HH practices. By contrast, a significant majority of 82% (n = 82) indicated that they had not received such training. Notably, second-year students received the highest percentage of training, which may reflect a targeted training initiative or curriculum component specific to that year. This distribution of training received is illustrated in Figure 3.

The overall knowledge of the respondents is 44%. Table 1 shows that a high level of awareness is evident in the understanding of the inappropriateness of using hand rubs on wet hands, with 80% of respondents demonstrating good knowledge in this area. However, there are significant gaps in other aspects of HH. For instance, while 65% of respondents correctly followed the steps in the hand-washing technique, a notable 35% were poor in this practice. Only 44% understood the minimal time required for the effectiveness of alcohol-based hand rubs, with the remaining 56% showing poor understanding. The knowledge of the differences between alcohol-based hand rubs and soap and water was particularly weak, as only 5% of the respondents correctly understood these differences, with 73% demonstrating poor knowledge. Furthermore, practices related to preventing transmission to HCWs and patients were mixed, with 27% and 14%, respectively, showing good practices, while a majority fell into the moderate category. Many respondents (49%) showed poor awareness of HH protocols.

**Table 1:** Knowledge assessment on hand hygiene practices among medical students

S.No	Questionnaire	Good (n(%))	Moderate (n(%))	Poor (n(%))
1	Avoidance associated with hand colonization	61 (61%)	0 (0%)	39 (39%)
2	Hand rub used on wet hands	80 (80%)	0 (0%)	20 (20%)
3	Minimal time for alcohol-based hand rub effectiveness	44 (44%)	0 (0%)	56 (56%)
4	Steps in correct hand washing technique	65 (65%)	0 (0%)	35 (35%)
5	Statements on alcohol-based hand rub vs. soap and water	5 (5%)	22 (22%)	73 (73%)
6	Preventing transmission to healthcare workers	27 (27%)	51 (51%)	22 (22%)
7	Preventing transmission to the patient	14 (14%)	61 (61%)	25 (25%)
8	Awareness of hand hygiene protocol	51 (51%)	0 (0%)	49 (49%)

The overall attitude of the respondents is 69%. Table 2 summarizes participant's attitudes toward hand washing. The study examined factors influencing HH among medical students, revealing that 33% would skip hygiene if colleagues did, while 67% would not. Material inadequacy hindered 60% from practicing hygiene, whereas 40% did not face this issue. Emergencies and other priorities impeded hygiene for 75%, in contrast to 25% unaffected by these factors. Despite 87% feeling knowledgeable about HH, 13% felt otherwise. Notably, 90% claimed adherence to proper hygiene practices, while 10% admitted inconsistency.

**Table 2:** Participants' attitude toward hand washing

S.No	Questionnaire	Good (n(%))	Poor (n(%))
1	I won't do hand hygiene as my colleague won't do.	33 (33%)	67 (67%)
2	Because of the inadequacy of material, I miss to perform hand hygiene.	60 (60%)	40 (40%)
3	Emergencies and other priorities make hygiene more difficult at times.	75 (75%)	25 (25%)
4	I have sufficient knowledge about hand hygiene.	87 (87%)	13 (13%)
5	I adhere to correct hand hygiene practices at all times.	90 (90%)	10 (10%)

Table 3 presents the hand-washing practices of HCWs across different situations. The data reveal a significant variation in adherence to hand-washing protocols. Hand washing before meals or snacks and after using the washroom sees higher compliance (n = 83), with the majority reporting they "Always" wash their hands in these scenarios. However, hand washing before and after patient contact exhibits lower consistent adherence (14%), with many HCWs washing their hands "sometimes" (n = 78) or "never" (n = 11) in these critical contexts.

**Table 3:** Hand-washing practices among medical students

Practice	Always (n(%))	Sometimes (n(%))	Never (n(%))
Frequency of hand washing before touching the patient	14(14%)	78 (78%)	11 (11%)
Frequency of hand washing before simple procedure	32 (32%)	55 (55%)	18 (18%)
Frequency of hand washing after procedure	39 (39%)	63 (63%)	8 (8%)
Frequency of hand washing after simple procedure	47 (47%)	59 (59%)	13 (13%)
Frequency of hand washing in a day’s procedure	52 (52%)	61 (61%)	1 (1%)
Frequency of hand washing before meals or snacks	83 (83%)	16 (16%)	0 (0%)
Frequency of hand washing after going to washroom	71 (71%)	27 (27%)	3 (3%)

## Discussion

This study aimed to assess the knowledge, attitudes, and practices (KAP) regarding HH among medical students at Saveetha Medical College. Our findings indicate a predominantly female participation (60%), compared to males (40%). This is consistent with a similar study by Jayarajah et al. [11], which reported a female-to-male participation ratio of 1:1.2. In our study, second-year students constituted the majority (44%), with third- and final-year students each making up 18% and first-year students accounting for 20%. However, in the study by Saxena et al. [12], the participants were mainly comprised of first-year students when compared to other years. The study revealed that the overall KAP toward HH among the students was moderate, with an average score of 53.52%. Specifically, knowledge scored 44%, attitude 69%, and practices 48.2%. While the students demonstrated a positive attitude toward HH protocols, their practices and knowledge were moderate. Notably, their scores on HH practices (48.2%) were higher than their knowledge scores (44%). These findings align with several other studies in the literature [13-15]. Possible reasons for the gap between knowledge and practice include a lack of emphasis on HH training in the curriculum, limited access to training programs, or insufficient awareness of HH among medical students. A critical knowledge gap identified was the lack of awareness regarding the necessity of performing HH before patient contact and after contact with the patient's surroundings. Addressing these gaps through educational interventions could improve compliance and understanding.

Our results were better compared to other studies in India, such as the one by Deepak et al. [6], which reported 51.5% awareness. Despite this, adherence to HH practices remains suboptimal, influenced by factors such as personal knowledge, perception of benefits, professional background, gender, severity of infectious diseases, work intensity, and the availability of facilities. An Indian study by Nair SS et al. [9] among dental students observed 98% sensitivity, the highest reported so far. Following the WHO guidelines on HH, it is recommended that alcohol-based hand rub be used for at least 20 seconds to effectively eliminate most germs. However, only 44% of our students answered this question correctly. By contrast, a study in Saudi Arabia by Abdulaziz Alotaibi et al. [1] reported a 52.6% correct response rate, while a study in India by Modi Pranav et al. [10] found only 36.1% answered appropriately. These findings highlight the need for additional education to reduce HCAIs. In our study, 25% of the participants reported that HH practices were not followed during emergencies. This is consistent with a study by Salama et al. [16], which found that 91.3% of HCWs did not follow HH practices in emergencies. Knowledge and attitude toward hand washing significantly influence its practice. This study also revealed that undergraduate students tend to perform HH more often after patient contact than before and more after bedside procedures than before.

In our study, 27% of the participants performed HH before touching a patient, while a study by Thompson et al. [17] reported a zero percent hand-washing rate before patient contact. Adherence to HH practices, whether through the use of water and soap or alcohol-based hand rub gel, is crucial in reducing healthcare expenditures globally [18]. In addition, 40% of the participants cited the lack of sinks, soaps, alcohol-based sanitizers, paper towels, and water as reasons for not performing HH, which aligns with the study of Naik TB et al. [19]. While the medical students at Saveetha Medical College show a moderate understanding of HH protocols and their importance in preventing nosocomial infections, there are evident gaps between knowledge and practice. According to WHO data, HCAIs represent a significant global health challenge, impacting millions of patients annually [20]. Studies have shown that effective HH practices can prevent 15-30% of total HCAIs [21]. Despite various strategies to improve HH, there remains a persistent gap in effective hygiene practices. Several factors influence HH adherence, including personal knowledge, perceived benefits, professional background, gender, severity of infectious diseases, work intensity, and the availability of facilities [20]. Addressing these gaps through structured training sessions and regular surveillance programs is essential. By focusing on these educational initiatives, adherence to HH protocols can be significantly improved, thereby enhancing the overall quality of healthcare services and reducing the incidence of HCAIs. The findings of this study provide a valuable baseline for future interventions aimed at improving HH practices among medical students and healthcare professionals.

Limitations of the study

Since it is a questionnaire-based study, it often relies on self-reported data, which can introduce bias due to social desirability or recall inaccuracies. The generalizability of results may be limited causing the risk of misinterpretation due to limited sample size. The cross-sectional nature of the study means that it captures data at one point in time, limiting the ability to infer causality or track changes in HH practices over time.

## Conclusions

Despite medical students at our institution demonstrating satisfactory knowledge of HH protocols, a notable gap exists between their understanding and actual practice. To bridge this gap, structured training sessions and practical hands-on experiences must be integrated into the medical curriculum, supported by surveillance programs to monitor compliance. Enhancing infection control education and fostering a culture that prioritizes HH can significantly improve adherence, ultimately leading to safer healthcare environments and better patient outcomes.
